# Supplementary material for: Defective Induction of IL-27-Mediated Immunoregulation by Myeloid DCs in Multiple Sclerosis
Source: Int J Mol Sci. 2023 Apr 28;24(9):8000. doi: 10.3390/ijms24098000 (PMC10179146; doi:10.3390/ijms24098000)
Supplement: Supplementary file 1 [file ijms-24-08000-s001.zip › ijms-2240306-supplementary.pptx]

## Slide 1
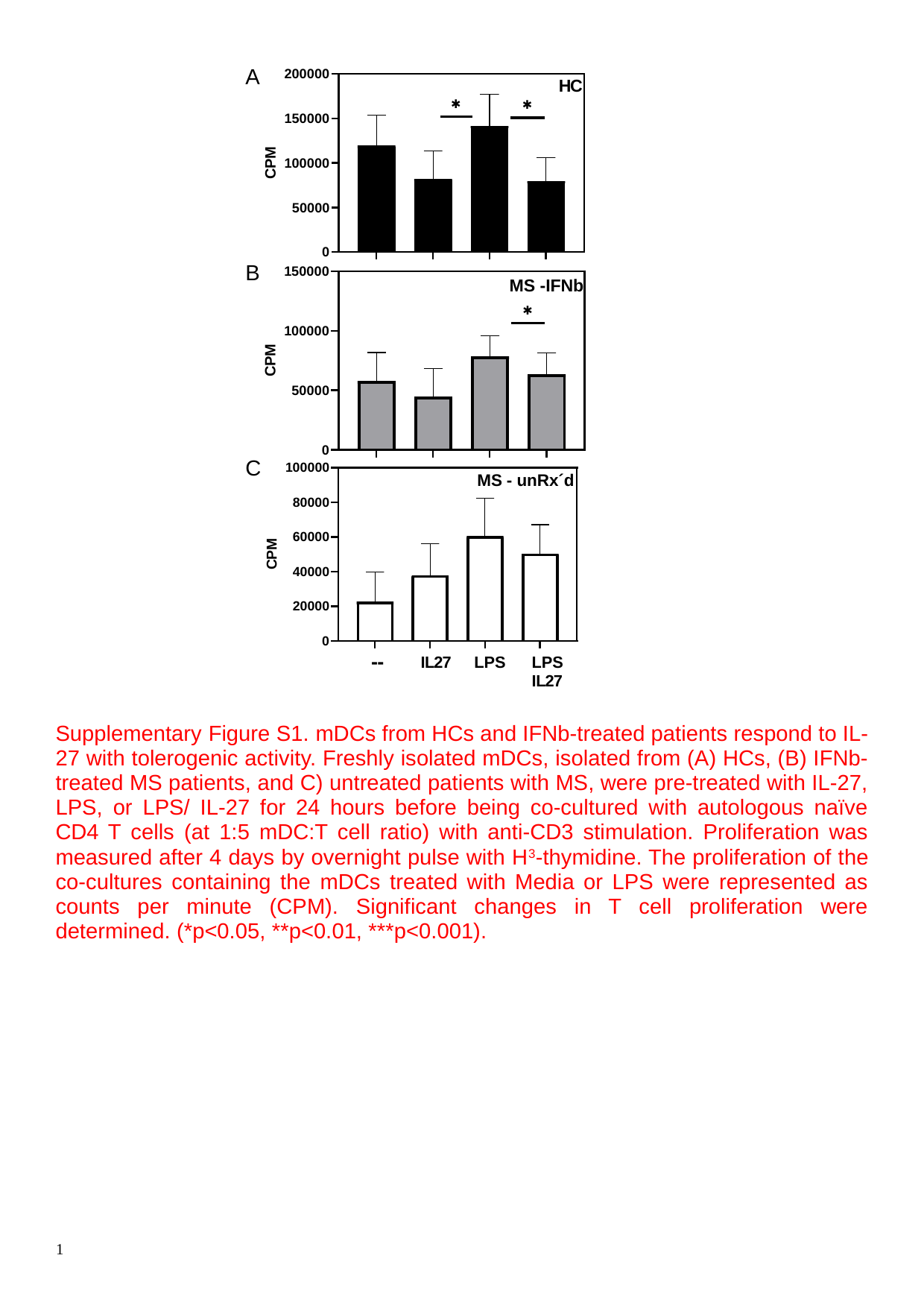

A
B
C
Supplementary Figure S1. mDCs from HCs and IFNb-treated patients respond to IL-27 with tolerogenic activity. Freshly isolated mDCs, isolated from (A) HCs, (B) IFNb- treated MS patients, and C) untreated patients with MS, were pre-treated with IL-27, LPS, or LPS/ IL-27 for 24 hours before being co-cultured with autologous naïve CD4 T cells (at 1:5 mDC:T cell ratio) with anti-CD3 stimulation. Proliferation was measured after 4 days by overnight pulse with H3-thymidine. The proliferation of the co-cultures containing the mDCs treated with Media or LPS were represented as counts per minute (CPM). Significant changes in T cell proliferation were determined. (*p<0.05, **p<0.01, ***p<0.001).
1

## Slide 2
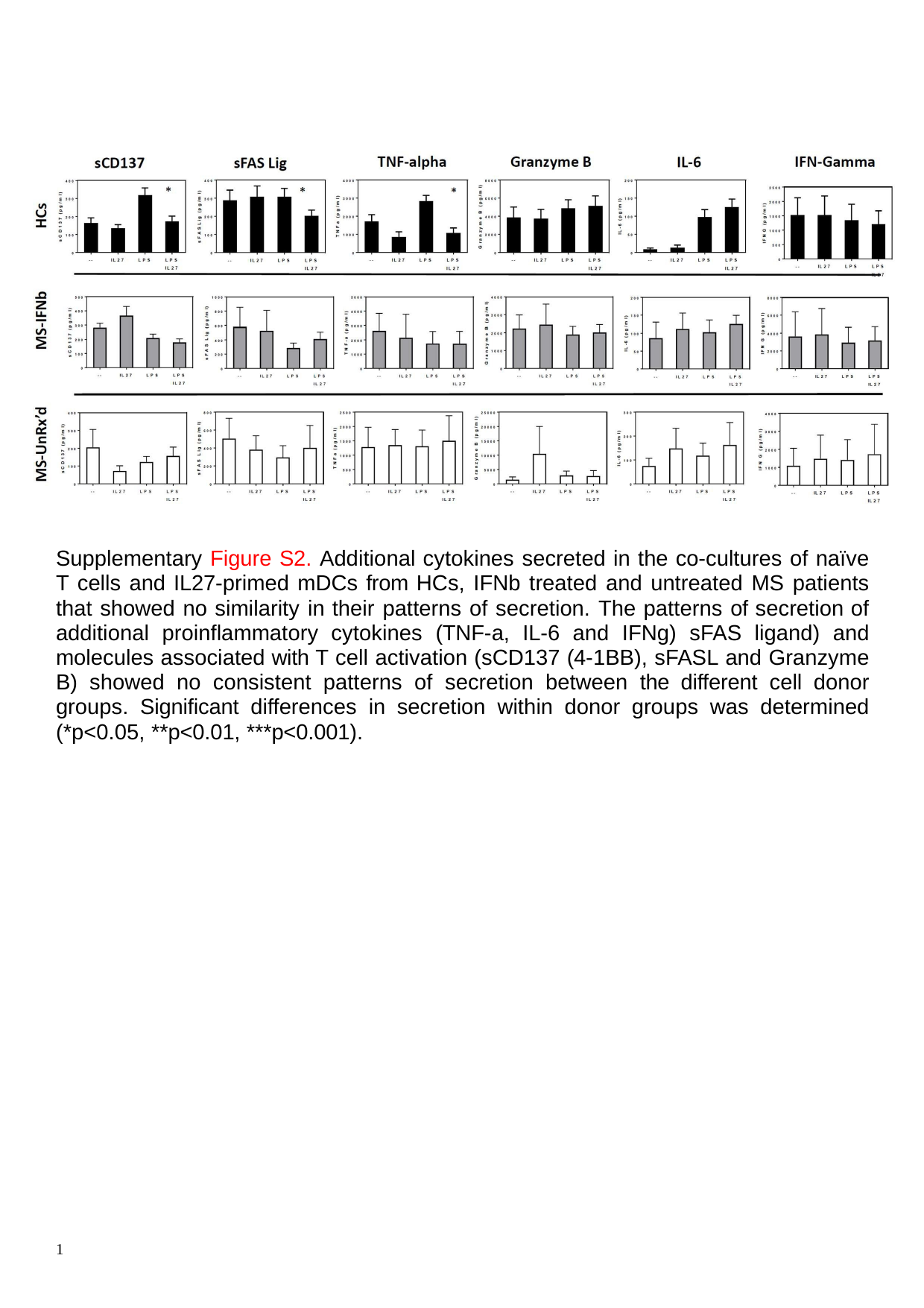

Supplementary Figure S2. Additional cytokines secreted in the co-cultures of naïve T cells and IL27-primed mDCs from HCs, IFNb treated and untreated MS patients that showed no similarity in their patterns of secretion. The patterns of secretion of additional proinflammatory cytokines (TNF-a, IL-6 and IFNg) sFAS ligand) and molecules associated with T cell activation (sCD137 (4-1BB), sFASL and Granzyme B) showed no consistent patterns of secretion between the different cell donor groups. Significant differences in secretion within donor groups was determined (*p<0.05, **p<0.01, ***p<0.001).
1

## Slide 3
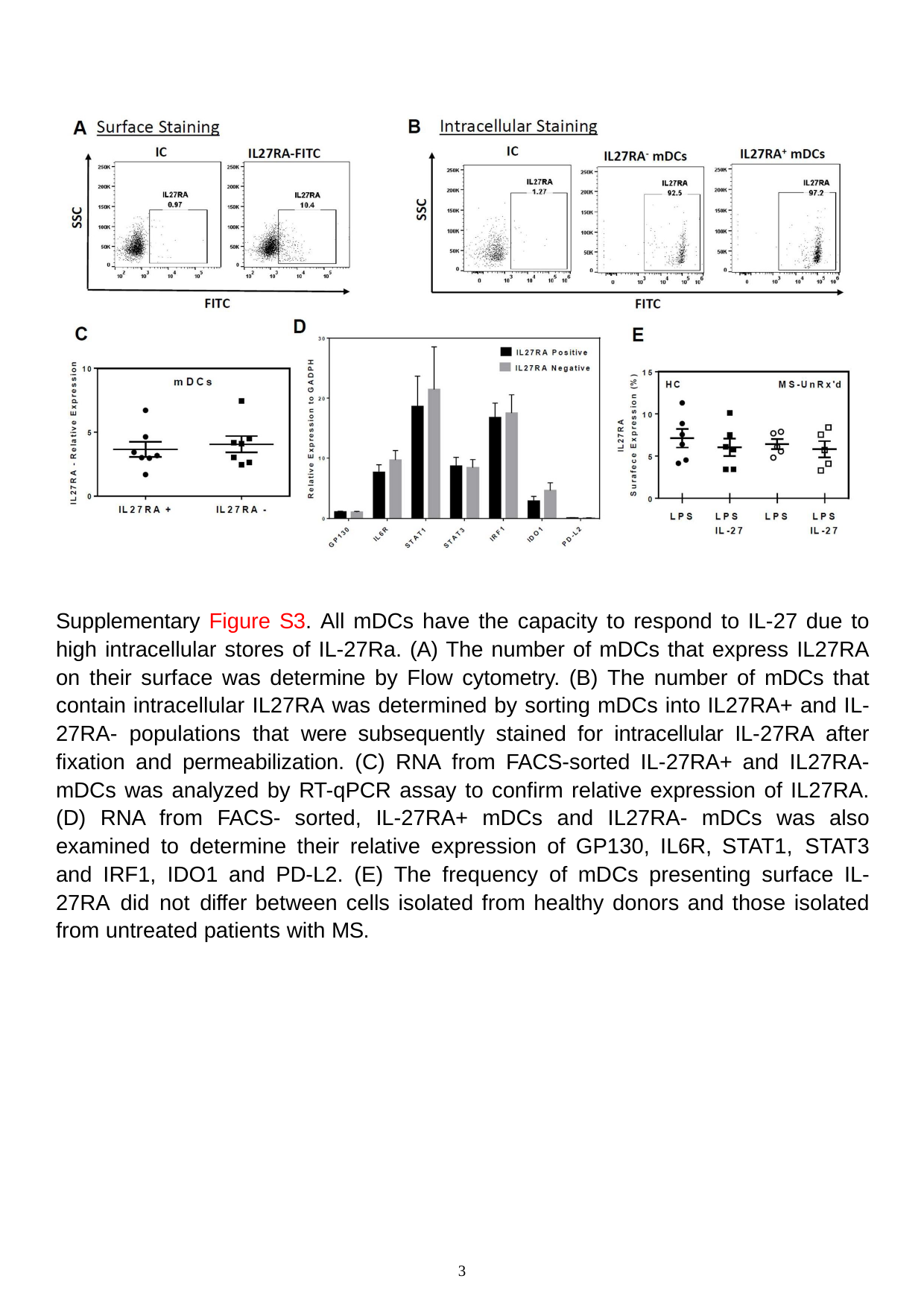

Supplementary Figure S3. All mDCs have the capacity to respond to IL-27 due to high intracellular stores of IL-27Ra. (A) The number of mDCs that express IL27RA on their surface was determine by Flow cytometry. (B) The number of mDCs that contain intracellular IL27RA was determined by sorting mDCs into IL27RA+ and IL-27RA- populations that were subsequently stained for intracellular IL-27RA after fixation and permeabilization. (C) RNA from FACS-sorted IL-27RA+ and IL27RA- mDCs was analyzed by RT-qPCR assay to confirm relative expression of IL27RA. (D) RNA from FACS- sorted, IL-27RA+ mDCs and IL27RA- mDCs was also examined to determine their relative expression of GP130, IL6R, STAT1, STAT3 and IRF1, IDO1 and PD-L2. (E) The frequency of mDCs presenting surface IL-27RA did not differ between cells isolated from healthy donors and those isolated from untreated patients with MS.
3

## Slide 4
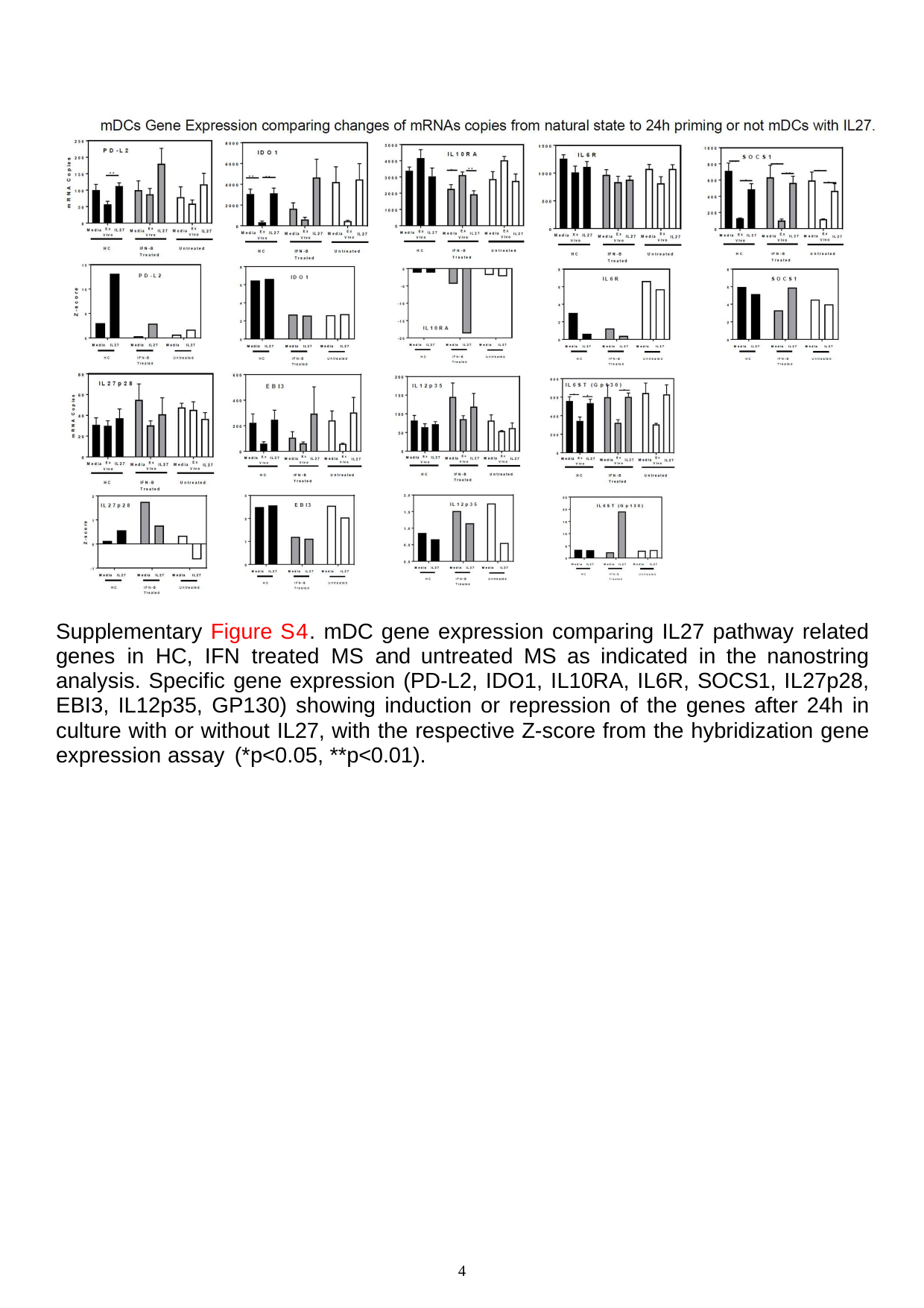

Supplementary Figure S4. mDC gene expression comparing IL27 pathway related genes in HC, IFN treated MS and untreated MS as indicated in the nanostring analysis. Specific gene expression (PD-L2, IDO1, IL10RA, IL6R, SOCS1, IL27p28, EBI3, IL12p35, GP130) showing induction or repression of the genes after 24h in culture with or without IL27, with the respective Z-score from the hybridization gene expression assay (*p<0.05, **p<0.01).
4

## Slide 5
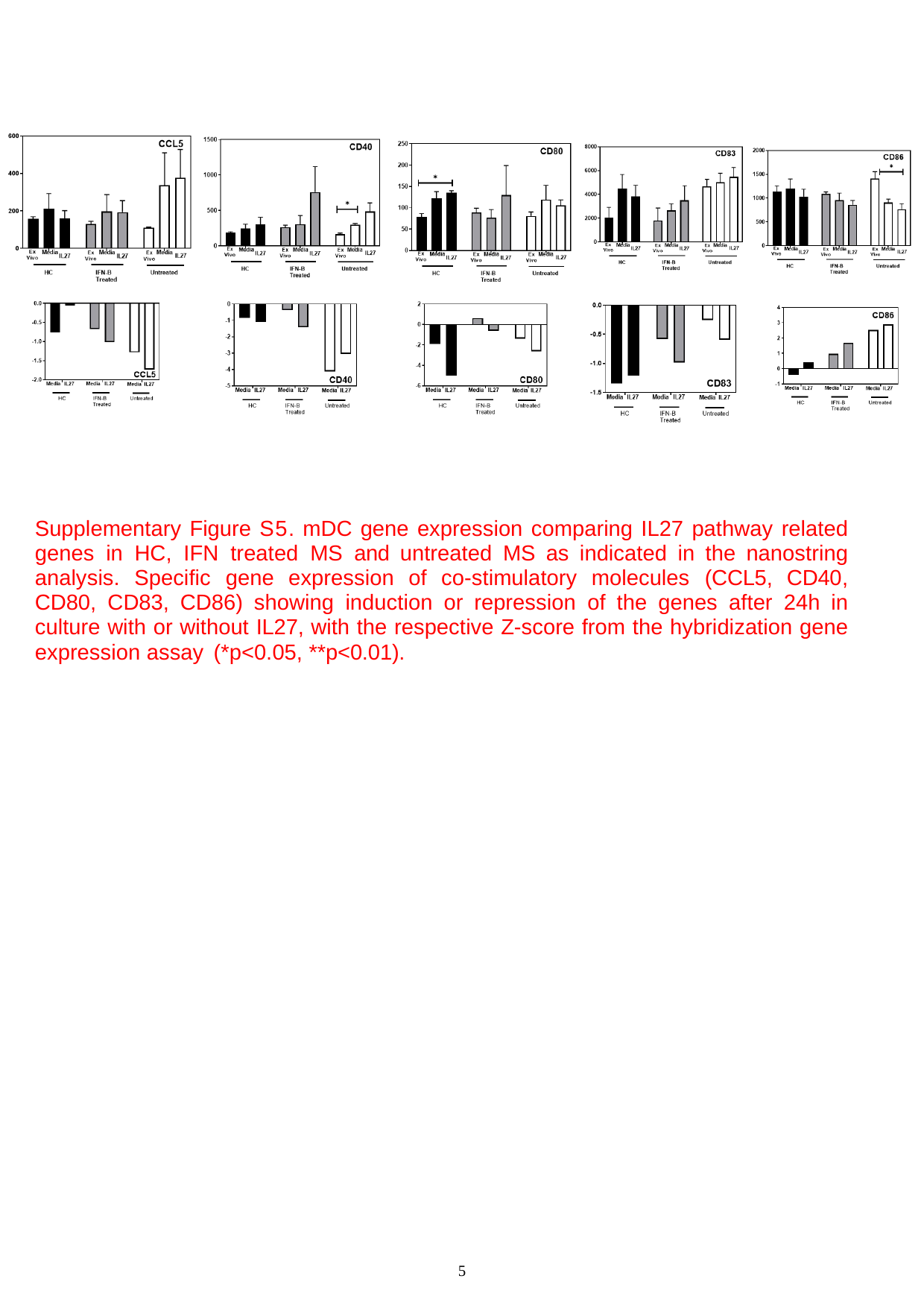

Supplementary Figure S5. mDC gene expression comparing IL27 pathway related genes in HC, IFN treated MS and untreated MS as indicated in the nanostring analysis. Specific gene expression of co-stimulatory molecules (CCL5, CD40, CD80, CD83, CD86) showing induction or repression of the genes after 24h in culture with or without IL27, with the respective Z-score from the hybridization gene expression assay (*p<0.05, **p<0.01).
5

## Slide 6
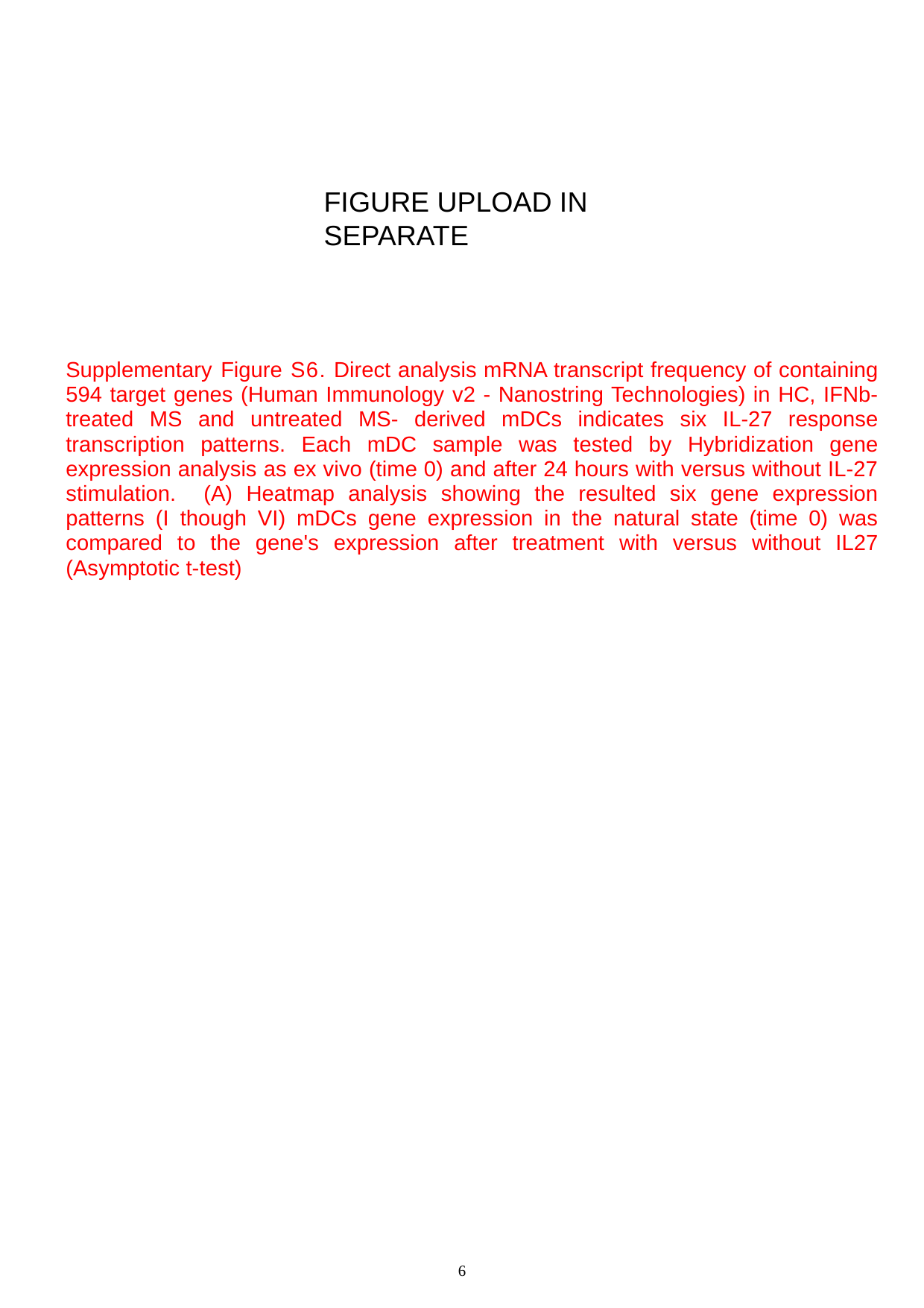

FIGURE UPLOAD IN SEPARATE
Supplementary Figure S6. Direct analysis mRNA transcript frequency of containing 594 target genes (Human Immunology v2 - Nanostring Technologies) in HC, IFNb-treated MS and untreated MS- derived mDCs indicates six IL-27 response transcription patterns. Each mDC sample was tested by Hybridization gene expression analysis as ex vivo (time 0) and after 24 hours with versus without IL-27 stimulation. (A) Heatmap analysis showing the resulted six gene expression patterns (I though VI) mDCs gene expression in the natural state (time 0) was compared to the gene's expression after treatment with versus without IL27 (Asymptotic t-test)
6
