# Supplementary figures and images for: Defective Induction of IL-27-Mediated Immunoregulation by Myeloid DCs in Multiple Sclerosis
Source: Int J Mol Sci. 2023 Apr 28;24(9):8000. doi: 10.3390/ijms24098000 (PMC10179146; doi:10.3390/ijms24098000)

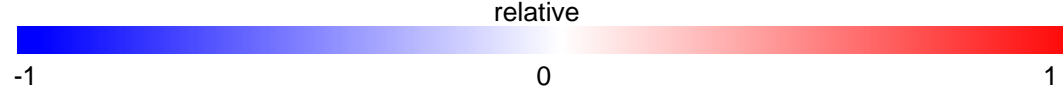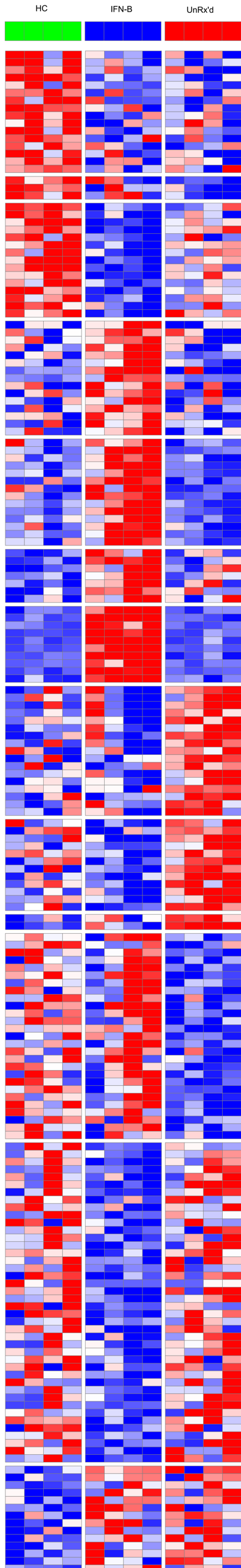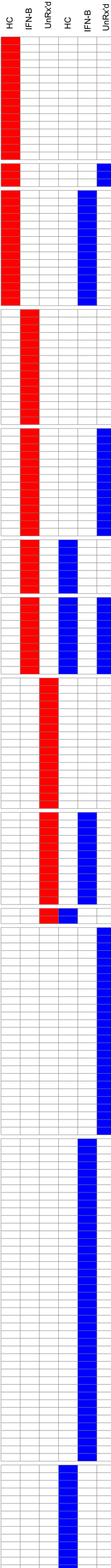

Supplement: Supplementary file 1 [file ijms-24-08000-s001.zip › Supplementary_6_HeatmapResults.pdf]
